# Short Stature in Moyamoya Disease: A Systematic Review of Potential Mechanisms and Clinical Outcomes

**DOI:** 10.1155/srat/5550395

**Published:** 2025-09-08

**Authors:** Abdallah M. Mujbel, Lea Nohra, Haidar Karrar T. Sabih, Rania H. Al-Taie

**Affiliations:** ^1^College of Medicine, Mustansiriyah University, Baghdad, Iraq; ^2^Faculty of Medical Science, Lebanese University, Beirut, Lebanon; ^3^Al Iraqia University College of Medicine, Baghdad, Iraq

**Keywords:** endocrine dysfunction, moyamoya disease, short stature

## Abstract

**Background:** Moyamoya disease (MMD) is a complex cerebrovascular disorder. While its neurological manifestations are well documented, the association between MMD and short stature remains underrecognized. This review explores potential mechanisms linking MMD with growth impairment, with a focus on endocrine and syndromic contributors.

**Methods:** A systematic review was conducted in accordance with PRISMA guidelines using PubMed and Scopus databases. Studies reporting cases of MMD with short stature or growth impairment were included. Data were extracted on patient demographics, endocrine findings, genetic mutations, neuroimaging, management, and outcomes. A narrative synthesis approach was used due to heterogeneity in study designs.

**Results:** Across 25 studies, 30 individuals with MMD and clinically significant short stature were identified, predominantly pediatric (2.5–52 years). Presentations frequently included seizures (*n* = 21), TIAs (*n* = 8), hemiparesis (*n* = 7), cognitive impairment (*n* = 8), and headaches (*n* = 3); in many, growth failure predated neurological events. Height deficits ranged from −2.13 to −23.7 SDS. Endocrine involvement was common: growth hormone deficiency (*n* ≈ 6), delayed bone age (*n* = 3), and other pituitary–thyroid–gonadal disturbances; a rare pituitary stalk duplication was reported. Management varied. Indirect revascularization in selected cases reduced recurrent ischemia; growth hormone therapy improved height velocity. Antiplatelets were commonly used; anticoagulation occasionally led to complications. Outcomes were heterogeneous; four deaths occurred, typically in patients with severe multisystem disease.

**Conclusion:** Growth retardation in MMD is generally a manifestation of hypothalamic–pituitary dysfunction, chronic cerebral hypoxia, or genetic syndromes. The observations in the present study suggest that MMD may be part of a more generalized multisystemic disorder in some patients and needs multisystemic assessment and management.

## 1. Introduction

Moyamoya disease (MMD) is a rare cerebrovascular disorder characterized by progressive stenosis of the intracranial internal carotid arteries and their branches, with the formation of fragile collateral vessels known as “moyamoya” [1]. It is most frequently encountered in East Asian populations, particularly in Japan, where it has an incidence of approximately 5 in 100,000 individuals, with a higher incidence in women [2].

MMD has two peak age groups of presentation: 5–10 years and 30–50 years [3]. The presentation varies with age. Cerebral hemorrhage commonly occurs in adults due to rupture of the weak moyamoya vessels, while children often present with ischemic events such as strokes, transient ischemic attacks (TIAs), headaches, and seizures [2]. Cognitive development, motor function, and psychosocial functioning may also be affected by the illness because of recurrent ischemic insults [1, 2]. The fundamental pathogenesis remains unclear, although genetic mechanisms, including mutations in RNF213, are involved, with a more prominent role of genetic factors in East Asians [4]. Recent studies also suggest a link between endocrine dysfunction and MMD, specifically growth hormone deficiency (GHD) and hypogonadotropic hypogonadism, indicating a broader systemic involvement beyond cerebral vascular disease [5].

Chronic cerebral hypoxia and hypothalamic–pituitary axis dysfunction have also been linked to growth failure in children, although the mechanisms remain unclear [6]. Although MMD is not treatable, early diagnosis and intervention, including revascularization surgery and medical management with antiplatelet therapy, can improve outcomes and lower the risk of ischemic or hemorrhagic events [1, 5, 6]. Despite greater awareness of MMD's systemic effects, significant gaps exist in understanding its influence on growth, especially short stature. The specific ways in which chronic cerebral hypoxia, hypothalamic–pituitary axis dysfunction, and multisystem impairments cause growth abnormalities are not well understood. Furthermore, reliable biomarkers for assessing disease severity, predicting prognosis, and guiding early intervention are lacking. Most current research mainly focuses on the cerebrovascular aspects of MMD, with limited investigation into its endocrine and systemic effects [1–6].

This systematic review is aimed at comprehensively analyzing the relationship between MMD and short stature, synthesizing evidence on potential pathophysiological pathways and contributing factors.

## 2. Methods

This systematic review was conducted following the PRISMA (Preferred Reporting Items for Systematic Reviews and Meta-Analyses) guidelines to ensure methodological quality, transparency, and reproducibility in study identification, data extraction, and synthesis [7]. An extensive and systematic search of PubMed and Scopus was performed. The search strategy terms were (“Moyamoya disease” OR “Moyamoya syndrome”) AND (“Short stature” OR “growth impairment”). The retrieved studies were imported into Rayyan, an online tool for systematic reviews, to facilitate blinded and independent screening by multiple reviewers. Duplicates were removed, and two independent reviewers conducted the title and abstract screening based on predefined inclusion and exclusion criteria. Discrepancies were resolved by discussion or consultation with a third reviewer. Full-text articles of potentially relevant studies were then retrieved and further assessed for eligibility.

### 2.1. Inclusion and Exclusion Criteria

The inclusion criteria for this systematic review were studies that had investigated the relationship of MMD with growth impairment, in the form of short stature. Studies that had reported endocrine or hormonal dysfunction, in particular hypothalamic–pituitary axis involvement, were also included. Studies that had reported clinical data in the form of case reports, observational studies, or clinical trials were included to obtain pertinent and high-quality evidence. Studies were excluded if they had not reported growth parameters or endocrine function and if they were reviews, commentaries, or articles that did not include primary data. Publications in non-English languages without available translations were also not included.

### 2.2. Quality Assessment and Risk of Bias

The methodological quality of the included studies was assessed using the CARE (Case Report) guidelines for case reports and case series, ensuring completeness and reliability of clinical data [8]. Two independent reviewers performed the quality assessment, with discrepancies resolved by consensus.

### 2.3. Data Extraction and Synthesis

Data extraction for this systematic review was conducted using a structured approach, capturing key study characteristics such as authors, year, study design, and sample size. Patient demographics, including age, sex, ethnicity, and clinical presentation, were systematically recorded. Growth parameters such as height, weight, BMI, and standardized growth charts were documented to assess the extent of growth impairment. Additionally, endocrine findings, including hormonal levels, GHD, and pituitary dysfunction, were extracted to explore potential endocrine involvement. A narrative synthesis approach was employed instead of a meta-analysis.

## 3. Results

### 3.1. Study Characteristics

This review included 25 studies comprising 30 patients diagnosed with both MMD and short stature. The studies spanned from 1984 to 2024 and originated from diverse geographic regions, including Japan, the United States, Spain, South Korea, and Taiwan. The majority of the studies were case reports (*n* = 22), with a few small case series (*n* = 3). The age of the affected individuals ranged from 2.5 to 52 years, with a predominance of pediatric cases (Table 1).

**Figure 1:**
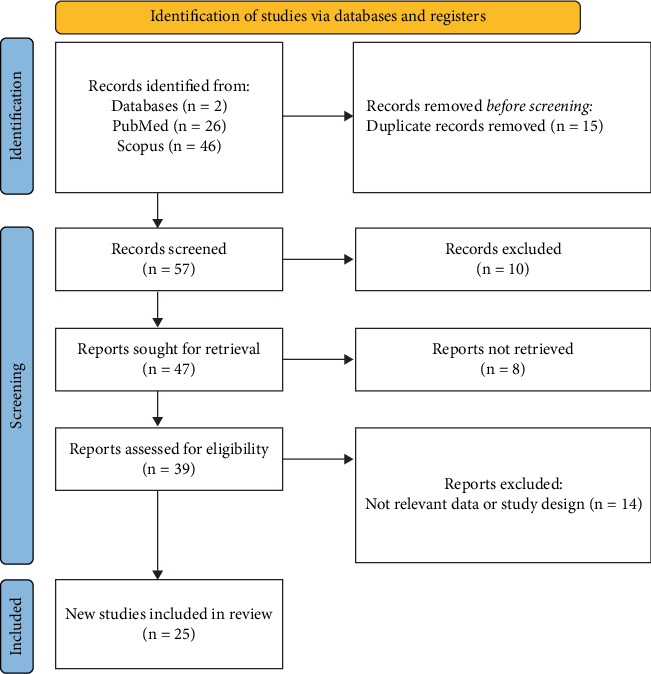
PRISMA flowchart of the included studies.

A total of 72 records were identified from PubMed (26) and Scopus (46). After removing 15 duplicates, 57 records were screened by title and abstract. Of these, 10 were excluded, and 47 full-text reports were sought. Eight reports could not be retrieved, leaving 39 articles for eligibility assessment. Fourteen were excluded due to irrelevant data or study design, resulting in 25 studies that were finally included in the review (Figure 1).

The quality assessment of the included studies revealed variability in methodological rigor. While most studies demonstrated adequate reporting of patient characteristics and interventions, some exhibited risk due to incomplete follow-up data and lack of standardized outcome measures (Table 2).

### 3.2. Demographic and Clinical Features

**Figure 2:**
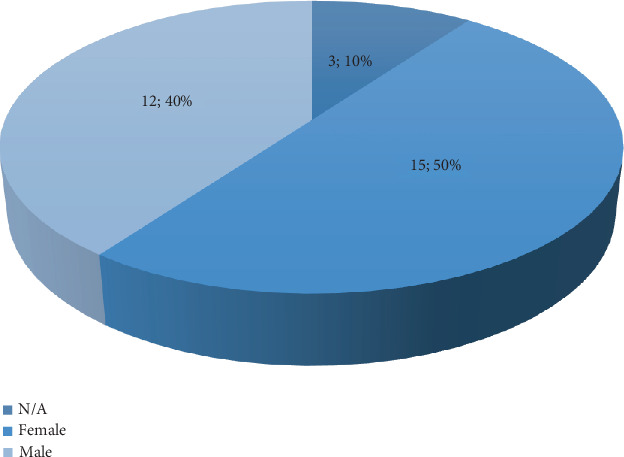
Gender distribution of the included patients (*N*, %).

Among the 30 patients, there were 12 males and 15 females (Figure 2). Many of these patients had comorbid conditions associated with their MMD and short stature. Notably, syndromic conditions such as Turner syndrome (*n* = 11), Noonan syndrome (*n* = 3), and microcephalic osteodysplastic primordial dwarfism type II (MOPD2) (*n* = 14) were present. Additionally, endocrinopathies such as hypothyroidism and GHD (*n* = 13) were reported.

The clinical presentation was highly variable. Common symptoms included TIA (*n* = 8), hemiparesis (*n* = 7), recurrent seizures (*n* = 21), cognitive impairment (*n* = 8), and recurrent headaches (*n* = 3). In many cases, patients had a prolonged history of growth failure before the onset of neurological symptoms.

### 3.3. Growth and Endocrinological Findings

Short stature was observed across all cases, with height standard deviation score (SDS) ranging from −2.13 to an extreme of −23.7. GH deficiency was diagnosed in six cases, with peak GH levels ranging from 0.55 to 2.6 *μ*g/L. Three patients had delayed bone age, which also indicated an underlying endocrine disorder. Other hormonal abnormalities, including inadequate gonadotropin levels, hypothyroidism, and hypergonadotropic hypogonadism, were also found in some patients.

### 3.4. Neuroimaging and Vascular Findings

Neuroimaging was performed in all cases with MRI and MRA, which diagnosed moyamoya vasculopathy. The most common radiological findings included stenosis or occlusion of the internal carotid artery (*n* = 18), formation of collateral circulation with a “puff-of-smoke” pattern (*n* = 17), and bilateral involvement of the middle cerebral artery (*n* = 7). Secondary features of progressive cerebral atrophy (*n* = 1), leptomeningeal anastomosis (*n* = 15), and ischemic infarctions or intracerebral hemorrhages (*n* = 11) were also reported. Apart from cerebrovascular disease, systemic imaging also revealed additional abnormalities, including renal malrotation and congenital heart disease in the form of atrial and ventricular septal defects. Skeletal dysplasia was also reported in a few patients.

### 3.5. Genetic Findings

Genetic testing was performed in seven cases, and pathogenic mutations in genes involved in syndromic growth disorders and cerebrovascular disease were identified. Mutations in SHOC2, which is involved in Noonan-like syndrome, were identified in two cases, and SMARCAL1 mutations associated with Schimke immuno-osseous dysplasia were confirmed in two additional cases. In a patient with Turner syndrome, there was chromosomal mosaicism with a 45,X/46,XX/47,XXX karyotype.

### 3.6. Medical and Surgical Management

The management of MMD in patients with short stature varied significantly across the 25 studies reviewed (Table 3). Medical management strategies included GH therapy (*n* = 5), aspirin therapy (*n* = 2), calcium channel blockers (*n* = 7), steroids (*n* = 16), and anticonvulsants such as carbamazepine (*n* = 10). GH therapy was prescribed in patients presenting with confirmed GHD or significant height impairment, with reported improvements in growth velocity in several cases. Aspirin was used primarily for secondary stroke prevention in patients with TIA or cerebrovascular occlusions. Other supportive medical treatments included antihypertensive agents (n = 16), bisphosphonates for osteoporosis (*n* = 16), and anticoagulation therapies such as warfarin and enoxaparin in selected cases (*n* = 23).

Surgical intervention was performed in select patients with progressive cerebrovascular disease. The most frequently employed procedure was encephaloduroarteriosynangiosis (EDAS) (*n* = 3), a revascularization surgery aimed at improving collateral blood flow to ischemic regions of the brain. Bilateral EDAS was performed in one patient with successful revascularization, while another patient underwent unilateral EDAS using the right superficial temporal artery. A single case of bilateral pial synangiosis surgery was reported. However, some patients were deemed poor candidates for surgical intervention due to advanced disease or underlying medical conditions (*n* = 3).

### 3.7. Complications and Their Management

Complications were documented in a subset of patients undergoing either medical or surgical treatment. One patient on aspirin therapy developed high fever, erythema, and bilateral leg wheals, necessitating discontinuation of the medication. Another patient on aspirin and warfarin developed a subdural hematoma, which led to cessation of anticoagulation therapy. A patient receiving GH therapy reported no adverse effects, while another demonstrated a significant increase in height velocity (12 cm in 1 year). In patients who underwent surgical revascularization, no major postoperative complications were reported, and there were no documented cases of surgical failure.

### 3.8. Follow-Up and Outcomes

Follow-up duration varied widely among studies, ranging from 6 months to over 18 years. Patients receiving GH therapy showed a tendency to improve growth parameters, with increases in height ranging from 4 to 12 cm over varying follow-up durations. On the other hand, some patients with widespread cerebrovascular involvement continued to experience frequent TIA or neurological deterioration despite treatment. In the surgically treated patients, successful revascularization was noted in several cases, with no further ischemic events on follow-up.

### 3.9. Mortality and Prognosis

Mortality was recorded in four patients, highlighting the potential severity of MMD in patients with complex medical backgrounds. The fatal cases included individuals with underlying Turner syndrome, nephrotic syndrome with steroid resistance, and severe cerebrovascular involvement leading to progressive neurological decline. Palliative management was provided in some cases, particularly for those with extensive cerebrovascular occlusion or associated systemic disease.

## 4. Discussion

This systematic review reveals a robust and underrecognized association between MMD and short stature. Our study demonstrates that short stature in MMD is not random but often reflects hypothalamic–pituitary axis dysfunction, cerebral hypoxia, or syndromic associations with multisystem involvement.

GHD was noted in several cases, with peak GH levels of 0.55–2.6 *μ*g/L, documenting disturbed somatotropic axis function. For instance, Kalina et al. [12] reported an adolescent boy with isolated GHD. Abdullah et al. [27] also reported two brothers with low IGF-1 levels and subnormal GH responses, further documenting this endocrine association. Besides isolated GHD, several patients had multiple endocrine disturbances. MacKenzie et al. [18] diagnosed panhypopituitarism in a 7-year-old boy with MMD, while Aljthalin et al. [21] reported thyroid and adrenal axis abnormalities. Choi et al. [15] and Byard et al. [19] reported cases of hypogonadotropic and hypergonadotropic hypogonadism, respectively. These findings introduce the concern that MMD-related ischemia can derange more extensive hypothalamic–pituitary function, either through direct vascular compromise or secondary neurodegeneration.

Consistent radiological findings across all studies included bilateral internal carotid artery stenosis, formation of moyamoya collateral vessels, and ischemic infarctions. Schuster et al. [11] confirmed progressive internal carotid artery narrowing via angiography, consistent with moyamoya vasculopathy. These patterns suggest chronic cerebral hypoxia as a plausible contributor to hypothalamic–pituitary injury and resultant growth hormone dysfunction. Hervé et al. [5] mentioned that cerebral hypoperfusion in hereditary MMD could impair neuroendocrine regulation through ischemic damage to the hypothalamus, a theory supported by imaging findings in several reviewed cases. In rare instances, structural pituitary anomalies such as pituitary stalk duplication have been observed. Sravya et al. [17] highlighted such a radiological finding in a 2.5-year-old girl, emphasizing the potential role of congenital pituitary malformations in endocrine dysfunction.

Revascularization procedures such as EDAS were performed in patients with progressive vascular compromise. Schuster et al. [11] documented successful bilateral EDAS with no recurrent TIAs. Kato et al. [25] noted cessation of ischemic events following surgical intervention using the superficial temporal artery. However, not all patients were surgical candidates due to underlying comorbidities or advanced disease. Growth hormone therapy was employed in patients with confirmed deficiency, with favorable outcomes. Kalina et al. [12] noted a 12-cm height increase in 1 year following GH administration. MacKenzie et al. [18] observed a 9.8-cm gain in height within a year of combined GH and thyroid hormone replacement.

A significant number of reviewed cases included syndromic diagnoses affecting growth and vascular development. Notably, Hervé et al. [5] mentioned that patients with hereditary moyamoya syndrome often exhibit systemic involvement, including pituitary hormonal disturbances, supporting the idea of MMD as a multisystem disorder rather than an isolated vasculopathy. Eslava et al. [9] described profound vascular changes alongside microcephaly and intellectual disability in a child with MOPD II. Furthermore, Manjila et al. [14] reported a patient with mosaic Turner syndrome, while Choi et al. [15] and Lo et al. [13] linked SHOC2 mutations to Noonan-like phenotypes with concurrent MMD. Lo et al. [13] emphasized the importance of SHOC2 mutations in connecting facial dysmorphia, loose anagen hair, and short stature with MMD. These findings reinforce that the short stature observed in many patients is not solely a result of cerebral ischemia but also a reflection of underlying syndromic and developmental pathology.

Aspirin is widely used in children with MMD who present with ischemic symptoms, serving as an antiplatelet agent to reduce the risk of thrombotic events. Its use is particularly common in the pre- and postoperative period to prevent stroke recurrence. However, anticoagulants such as warfarin are generally not favored in the pediatric population due to the increased risk of hemorrhage and lack of strong supporting evidence in MMD management [32, 33].

Surgical revascularization remains the cornerstone of treatment for symptomatic patients. In children, indirect revascularization procedures, such as EDAS and encephalomyosynangiosis, are preferred due to the smaller size of cerebral vessels and their capacity for developing collateral vessels. These procedures are aimed at promoting neovascularization over time. Direct procedures, such as superficial temporal artery to middle cerebral artery (STA-MCA) bypass, are more technically demanding in children and are thus less commonly performed in younger age groups [34, 35].

## 5. Limitations and Future Directions

While this review identifies a clear association between MMD and short stature, the evidence is largely limited to case reports and small series. Heterogeneity in diagnostic workups, hormonal testing, and outcome reporting limits the generalizability of conclusions. Future studies should prioritize standardized hormonal screening in pediatric MMD, prospective tracking of growth and endocrine function, and genetic testing in syndromic cases.

## 6. Conclusion

This systematic review reveals a novel and underrecognized association between MMD and short stature, often reflecting underlying hypothalamic–pituitary axis dysfunction, chronic cerebral hypoperfusion, and syndromic comorbidities. Short stature in these patients is not incidental but may serve as an early clinical marker of broader neuroendocrine or genetic involvement.

The consistent findings of GHD, delayed bone age, and structural pituitary abnormalities, along with improvements following hormone therapy, highlight the need for routine endocrine screening in pediatric patients with MMD. Additionally, the overlap with genetic syndromes suggests shared developmental pathways that warrant further exploration.

This review offers a new perspective on moyamoya as a multisystem disorder and calls for prospective studies to guide integrated endocrine, genetic, and neurovascular management in affected children.

## Figures and Tables

**Table 1:** Study characteristics and patient data.

**ID**	**Author**	**Year published**	**Country**	**Study design**	**Sample size**	**Age (year)**	**Gender**	**Comorbidity**	**Symptoms**	**Duration of symptoms (months)**	**Examination findings**	**Investigations**	**Laboratory findings**	**Imaging findings**
1	Eslava et al. [9]	2021	Spain	Case report	1	10	Male	NA	Short stature, intellectual disability, nasal voice	NA	Extremely short stature (−11.1 SD), marked microcephaly, retrognathia, dysplastic auricles, prominent nose with elevated nasal root and wide bridge, dental malposition and cavities	Laboratory tests: Mild thrombocytosis (616,000/*μ*L), hypernatremia (sodium 150 mEq/L), GH peak 0.93 ng/mL indicating deficiency, normal cortisol. Neuropsychological: WNV score similar to an 18-month-old	NA	MRI: Moyamoya syndrome with secondary brain atrophy, hypoplasia of the corpus callosum and anterior commissure. Abdominal ultrasound: Malrotation of the left kidney. X-rays: Osteoporosis, bilateral coxa vara, diffuse platyspondyly

2	Ishiguro et al. [10]	2001	Japan	Case report	1	6.5	Male	Cardiofaciocutaneous (CFC) syndrome	Left hemiplegia, right-sided eye deviation, hypotonia, failure to thrive, developmental delay, horizontal nystagmus, febrile seizures, TIA episodes	TIA episodes lasted 1–2 h each, other symptoms present since infancy	Hypoplasia of the supraorbital ridges, thin eyebrows, left ptosis, downward-angled ears, hyperkeratotic lesions, sparse hair, depressed nasal bridge, height (96.8 cm; −23.7 SD), head circumference (55.5 cm; −12.0 SD), Levin II/VI systolic murmur, hepatosplenomegaly, hyperreflexia in the left lower limb	Serum levels of liver enzymes, lactate, pyruvate, and amino acids were normal Normal coagulation system results	Liver enzymes, lactate, pyruvate, amino acids, coagulation profile: Normal; ECG: Incomplete right bundle branch block; cardiac ultrasound: Atrial septal defect, mild hypertrophy, normal function	MRI showed flow voids in both basal ganglia without infarction. MRA revealed bilateral ICA stenosis at the circle of Willis and enlarged perforating vessels Cerebral angiography confirmed occlusion of both middle cerebral arteries. EEG showed semicontinuous theta or delta waves in the right centroparietal areas

3	Schuster and Roberts [11]	1999	United States	Case report	1	7	Female	Noonan's syndrome, aortic coarctation	Recurrent headaches, nausea, transient right-sided weakness, sensory loss, slurred speech	12 months	Blood pressure discrepancy (between upper and lower limbs, mild left ventricular hypertrophy, mildly dysplastic mitral valve, mild supravalvular aortic narrowing)	MRI/MRA	Cardiac evaluation: Cardiac catheterization confirmed aortic coarctation with a significant pressure gradient Echocardiogram showed mild left ventricular hypertrophy and valvular abnormalities	MRI/MRA revealed bilateral distal carotid stenosis with collateral circulation consistent with MMD Cerebral angiography showed occlusion of the MCA and high-grade stenosis of the ACA bilaterally

4	Kalina et al. [12]	2004	Poland	Case report	1	15.4	Male	Retractile testes	Stabbing headaches (left parietotemporal region), nausea, hypersensitivity to light and sounds, fatigue, sleepiness, intense yawning	Symptoms started at 14 years old, ongoing	Height of 146 cm, classified as −4.3 SDS below the mean for age Headaches: Reported as a primary concern leading to the referral Facial dysmorphia: Discrete facial features that may suggest underlying conditions Bruxism Temporomandibular joint luxation Cryptorchidism Delayed sexual development	Hormonal assessments: Isolated GHD	Basal and stimulated hormonal assays: Low GH (max 2.6 *μ*g/L), no other pituitary hormone insufficiencies; psychological evaluation: IQ = 93, depression	MRI: Suggested malformation of cerebral vessels Angio CT and panangiography: Confirmed findings

5	Lo et al. [13]	2015	Taiwan	Case series	2	Case 1: 9 Case 2: 19	Case 1: Female Case 2: Male	Global developmental delay, short stature, nystagmus	Both patients exhibited significant growth retardation Distinctive facial features typical of Noonan-like syndrome were observed Patients had loose anagen hair, which is easily pluckable and sparse	Symptoms of developmental delay since infancy	Case 1: Height: 114.9 cm (−3.22 SDS), weight: 19 kg (−1.79 SDS), loose anagen hair, dysarthria, nystagmus, dysmorphic features Case 2: Height: 147.4 cm (−3.98 SDS), weight: 34.6 kg (+2.29 SDS)	Genetic testing: Heterozygous germline mutations in the SHOC2 gene were identified in both patients, linking their symptoms to Noonan-like syndrome	Case 1: Bone age: 6 years 10 months, IQ: Verbal 60, performance 55, full scale 54 (WISC-III); cardiac: 4.4-mm atrial septal defect, secundum type	MRI was utilized to confirm the presence of MMD, characterized by stenosis of the internal carotid arteries and collateral circulation

6	Manjila et al. [14]	2013	United States	Case report	1	52	Female	Mosaic Turner syndrome, short stature, pectus excavatum, small fingers, micrognathia	SAH and an ICH in the left frontal lobe	NR	Short stature (147 cm), pectus excavatum, small fingers, micrognathia	CT: ICH in the left frontal lobe CT angio: Right ICA moyamoya changes with hypertrophied lenticulostriate vessels, 4-mm unruptured superior hypophyseal aneurysm	Chromosomal mosaicism: 45,X (39%), 46,XX (9%), 47,XXX (52%)	Angiography: Occluded left intracranial ICA, hypertrophied lenticulostriate vessels around right ICA terminus, unruptured 4-mm aneurysm from the right superior hypophyseal artery. Chest CTA: Aberrant right subclavian artery, left vertebral artery from the aortic arch

7	Choi et al. [15]	2015	Korea	Case report	1	10	Female	Neonatal small atrial and ventricular septal defects (closed spontaneously)	Profound short stature and ectodermal abnormalities (sparse hair) At age 10, the patient experienced recurrent left hemiplegia TIAs (TIAs)	TIA at 10.2 years	Height: 104.9 cm (−2.62 SDS), weight: Not provided, head circumference: 56 cm (3.44 SDS)	Narrowing of both ICAs and both middle cerebral arteries Identified moyamoya-like vessels as collateral circulation	Genetic testing: Confirmed the presence of a mutation in the SHOC2 gene	Occlusion or narrowing of both ICAs and both MCAs Identified moyamoya-like vessels as collateral circulation

8	Yamashita et al. [16]	2004	Japan	Case report	1	12	Female	Antiphospholipid syndrome, Noonan syndrome	TIAs, cognitive difficulties, migraine-like headaches Symptoms associated with Noonan syndrome may include growth delays, distinctive facial features, and potential cardiac issues	6 months	Height: 120.8 cm (−5 SD), weight: 27 kg (−2 SD), multiple purpura lesions, dysarthria, chorea, hypotonia	Cranial MRI: Normal, cranial MRA: Moyamoya-like vascular changes, CT: Mild	Blood tests: Evaluations for antiphospholipid antibodies to confirm the diagnosis of antiphospholipid syndrome	MRA (MRA): Used to visualize the cerebral vasculature and confirm the presence of moyamoya-like changes

9	Sravya et al. [17]	2023	India	Case report	1	2.5	Female	NA	Short stature Micropenis Unilateral undescended testis Delayed motor milestones	Presenting at 2.5 years	Height: 84 cm (< third centile, −2.13 SD), weight: 12 kg (10–25th centile), midparental height: 168 cm, micropenis (2 cm stretched penile length)	Euthyroid status Suboptimal stimulated GH levels Suboptimal stimulated gonadotropin levels Normal cortisol and prolactin levels MRI of the pituitary showed pituitary stalk duplication	GHD confirmed	Pituitary stalk duplication A single pituitary gland of normal dimensions Fusion of the tuber cinereum and mammillary body

10	MacKenzie et al. [18]	1989	Sheffield	Case report	1	7	Male	Obesity, developmental delay	Short stature. The boy experienced generalized convulsions 6 months after starting hormone replacement therapy	Presenting at 7 years	Height: Well below the 3rd percentile (−3.1 SD), moderately obese, small penis, testes < 1 mL volume	Neuroradiological investigations were conducted, which typically include imaging techniques such as MRI or CT scans to assess brain structure and blood flow	Cortisol levels: 456 nmol/L at 0 min, abnormal thyroid function, raised prolactin, hypopituitarism confirmed	Cerebral angiography: Bilateral ICA occlusion, moyamoya syndrome

11	Byard [19]	2016	Australia	Case report	1	42	Female	Turner syndrome	Noonan-like syndrome symptoms included the following: Short stature Characteristic facial phenotype Sparse, thin hair	NA	Short stature (158 cm), webbed neck, underdeveloped breasts, increased carrying angle of the arm	Heterozygous germline mutations in the SHOC2 gene were identified in both patients	Bicuspid aortic valve, immature internal genitalia	CT/MRI: Flattening of gyri, SAH, ICH

12	Nishumora et al. [20]	2003	Japan	Case report	1	4.5	Male	NA	Left hemiparesis, generalized convulsions, propensity to fall, occasional mutism	From birth	Microcephaly, osteodysplastic features, primordial short stature, multiple café-au-lait spots	Genetic testing to confirm the diagnosis Imaging studies to assess cerebral vasculopathy Clinical evaluations to document physical symptoms and growth parameters	Radiological signs of MOPD2, multiple cafe-au-lait spots	Progressive stenosis of the internal carotid arteries and their branches

13	Aljthalin et al. [21]	2024	Saudi Arabia	Case report	1	14	Female	- Hypothyroidism - GHD - Seizures - Family consanguinity	Hemiparesis, recurrent episodes of right-sided weakness, short stature, seizures, migraine, unilateral headache, right-sided weakness	8 years (symptoms started at 6 years old)	- Right hemiparesis and hypertonia - Reflexes +3 in the right upper and lower limbs - Spastic gait - Head circumference at the 2nd percentile	MRI brain: Reduced adenohypophysis size, cerebral angiogram: Stenosis in the right ICA, moyamoya vessels	- TSH: 7.8 mIU/L (high) - ACTH: 0.9 pmol/L (low) - GH: 10.14 pmol/L (low) - IGF-1: 5.19 nmol/L (low) - EEG: Intermittent left hemisphere activity	- Brain MRI: Reduction in adenohypophysis size - Cerebral angiogram: Multiple stenoses in the right ICA, collateral circulation from posterior cerebral arteries, moyamoya vessels - MR angiography: Narrowing of the suprasellar portion of ICA (bilaterally more on the left)

14	Young et al. [22]	2004	United Kingdom	Case report	1	6.9	Female	MOPD2, café-au-lait spots	Intellectual disability Growth retardation	From birth	Height 84 cm, weight 10.4 kg, head circumference 42.8 cm (below the 3rd percentile)	Chromosome microarray analysis, which helped characterize the deletions associated with the phenotype Deletions ranged from 490 kb to 20.95 Mb within chromosome bands 1q23.3-q31.2	NA	Cerebral angiography: Extensive bilateral ICA narrowing with moyamoya collateral vessels

15	Hervé et al. [5]	2010	Algeria	Case report	1	22 years (onset of arm weakness), 34 years (age at death)	Male	Dilated cardiomyopathy, partial GH deficiency, hypergonadotropic hypogonadism, low cardiac output	Sudden right arm weakness, recurrent neurologic deficits, dyspnea, heart failure episodes, transient unconsciousness, seizures	(12–16 years)	Facial dysmorphism (mild bilateral ptosis, long philtrum, retrognathia, white hair locks), small hands with short, broad fingers, height of 1.52 m (−3.9 SD)	Multiple examinations such as GH tests, MRI, MCA	GHD, hypergonadotropic hypogonadism, azoospermia, decreased testicular volume (2.6 mL)	MRI: Right frontal cortical infarct, bilateral subcortical infarcts, new brain infarctions in MCA territory. Conventional angiography: ICA occlusion, moyamoya vessels, leptomeningeal anastomosis

16	Govender et al. [23]	2019	South Africa	Case report	1	7	Female	Nephrotic syndrome, epilepsy, osteoporosis	Short stature Development of steroid-resistant nephrotic syndrome Onset of moyamoya syndrome General signs of cellular immune insufficiency	From birth	Weight and height < –3 SD, platyspondyly, anterior vertebral beaking, small deformed femoral epiphyses	Genetic testing, which confirmed the presence of the c.1439 C>T mutation in the SMARCAL1 gene, a mutation known to be associated with SIOD	Genetic testing	MRA: Occlusion of posterior cerebral arteries, moyamoya pattern

17	Bang et al. [24]	2013	United States	Case report	1	5	Male	- Patent ductus arteriosus with pulmonary hypertension - Syndactyly of hands and feet - Developmental delay—Short stature - Dental enamel hypoplasia - Cutis marmorata - Microcephalic osteodysplastic primordial dwarfism type II (MOPD II) suspected	Decreased vision in the left eye, short stature, developmental delay, dental enamel hypoplasia	NA	- Visual acuity: 20/25 (right eye), 20/100 (left eye) - Macrovessel in the left iris with segmental nonperfusion - Old vitreous hemorrhage in the left eye - Peripheral retinal vascular leakage, nonperfusion, and telangiectasis in both eyes	Imaging studies	Genetic consultation suggested MOPD II due to intrauterine growth restriction	- MRI with angiography: Small left cerebral hemisphere, attenuated left ICA branches - MRA: Occlusion of supraclinoid left ICA, enlarged lenticulostriate and thalamostriate arteries with “puff-of-smoke” appearance

18	Kato et al. [25]	1984	Japan	Case report	1	13	Male	- Cutaneous syndactyly - Brachydactyly - Mental retardation (IQ 23) - Primary dwarfism - Hypertension (160/110 mmHg) - Vesicoureteral reflux	Weakness in the left upper extremity, inability to open the mouth, transient left hemiparesis, spastic paralysis of the left arm	24 months (since first hemiparesis at age 10)	- Left hemiparesis episodes (age 10, 11, and 12) - Spastic paralysis of the left arm - Inability to open the mouth (likely trigeminal nerve paralysis) - Increased tendon reflexes - Short stature (height: 120.7 cm, −4 SD)	EEG: 2–3 Hz high-voltage slow waves, CT scan, and vesicoureteric reflex	- Normal blood chemistry, renal function, and hormone tests - Chromosomal karyotype normal - GH normal	- Carotid angiography: Complete obstruction of supraclinoid internal carotid arteries bilaterally with moyamoya vascular network - CT: Multiple low-density areas in frontal–temporal regions - EEG: Slow waves in the right posterior quadrant suggestive of MMD

19	Vujic et al. [26]	2023	Serbia	Case report	1	6	Female	- Intrauterine growth retardation - Disproportionate short stature - Spondyloepiphyseal dysplasia - Triangular face, broad nasal tip, sparse hair - Absent dentition - TIA and partial epilepsy	Spastic weakness and seizures	23 months (symptoms started at 25 months, deceased at 5 years 9 months)	- Dysmorphic features: Short neck, triangular face, hyperpigmented macules - Aphasic episodes, tremors, left-sided spastic weakness - Contractures in lower extremities leading to loss of ambulation	Genetic test: Biallelic, nonsense mutation in SMARCAL1 gene EEG: Delta dysfunction, partial epilepsy diagnosis	- Normal GH and thyroid function - Severe lymphopenia - Negative tests for congenital/acquired thrombophilia - Proteinuria (at 30 months), hypercholesterolemia (at 31 months) - Normal renal function	- Initial MRI at 2.5 years: Brain atrophy, ischemic leukoencephalopathy, subcortical laminar necrosis - MRA: Absence of flow in the right MCA - Follow-up MRI/MRA: Worsening atrophy, bilateral chronic subdural hematomas, bilateral MCA and ACA occlusion/stenosis

20	Abdullah et al. [27]	2022	Iraq	Case report	2	Case 1: 13; Case 2: 10	Males	MMD, short stature	Abnormal body movement with recurrent convulsions	Case 1: 24 months (from onset at age 11–13) Case 2: 18 months (from onset at age 8.5–10)	Case 1: Dysmorphic facies (hypertelorism, saddle nose, broad forehead, low-set ears), weight: 42 kg (−0.65 SD), height: 141 cm (−2 SD), Grade 3 power in both upper and lower limbs, poor height gain, bone age: 11 years Case 2: Dysmorphic facies (same as older brother), weight: 31.5 kg (−0.27 SD), height: 130 cm (−1.4 SD), Grade 3 power in both upper and lower limbs, poor height gain, bone age: 8 years	MRI, CT, CBC was normal, decreased GH	Case 1: Normal CBC, renal function, serum calcium. Free T4: 1.5 mg/dL, TSH: 4.18 mIU/mL, IGF-1: 38 ng/mL, peak GH: 0.77 ng/mL Case 2: Normal CBC, renal function, serum calcium. Free T4: 1.47 mg/dL, TSH: 3.38 mIU/mL, IGF-1: 94 ng/mL, peak GH: 0.55 ng/mL	Case 1: MRI: Bilateral foci of abnormal signal in deep white matter and cortical/subcortical regions. MRA: Severely attenuated MCAs and ACAs bilaterally, extensive collateral vessels in the basal ganglia and hypothalamus Case 2: MRI: Bilateral anterior subcortical abnormal signal in the frontal and upper parietal lobes, acute infarction, small tortuous hyperintense foci in the temporal basal ganglia and parietal white matter

21	Gburek-Augustat et al. [28]	2020	Germany	Case report	3	Case 1: 5 years (onset of symptoms), 9 years (diagnosis) Case 2: 3 years (onset of headaches), 4 years (seizures and diagnosis) Case 3: 3 years (diagnosis)	Case 1: NA Case 2: NA Case 3: NA	Case 1: Genetically confirmed Noonan syndrome Case 2: Bacterial pneumonia at age 4 Case 3: Genetically confirmed microcephalic osteodysplastic primordial dwarfism type II (MOPD2)	Case 1: Focal epileptic seizures Case 2: Recurrent, severe headaches; cerebral seizures Case 3: NA	Case 1: 48 months (4 years) Case 2: 12–15 months Case 3: NA	Case 1: No further neurological abnormalities Case 2: NA Case 3: Microcephaly, very small stature	Radiological examinations such as MR and MRA	Case 1: NA Case 2: NA Case 3: NA	Case 1: MRI/MR angiography: Nonvisualization of M1/M2 branches of ACM, corkscrew-like vessels in the basal ganglia, ivy sign on T1WI and FLAIR Case 2: Bilateral occlusion of ACM, pronounced vascular collaterals, ivy sign on retrospective evaluation Case 3: MR angiography at 3 years: Progressive signal increase suggestive of MMD

22	Kilic et al. [29]	2012	Turkey	Case report	1	3 years	Female	MOPD II	Hemiparesis, seizures, cognitive impairment	18–36 months	Marked intrauterine growth deficiency, short stature, small genitalia, short limbs, clinodactyly, microcephaly, micrognathia, prominent nose, hypoplastic alae nasi, prominent eyes, left-sided hemiparesis	Karyotype, molecular analysis, MRA	Normal routine tests, normal karyotype (46,XX), molecular analysis revealed a homozygous splice site mutation in the PCNT gene (c.2609+1 G>A, intron 14)	MRA: Occlusion of the right ICA, severe stenosis in the left ICA, weak signals in bilateral anterior and middle cerebral arteries

23	Spengos et al. [30]	2006	Greece	Case report	1	47 years	Female	Hypertension, cataract, primary amenorrhea, Turner's syndrome	Left-sided hemiparesis, severe hemineglect	6 days	Small stature, left hemiparesis, severe hemineglect	Karyotyping, MRA/MRI	Karyotype: 45,X/46,X,i(Xq)	MRI: Multiple ischemic lesions in the right hemisphere (both cortical and subcortical). MRA: Hypoplastic common carotid arteries, bilateral ICA obstruction

24	Strong et al. [31]	2021	United States	Case report	2	Case 1: 17 months Case 2: 21 months	Case 1: Female Case 2: Male	Case 1: Skin disease, liver and kidney issues, hypertension Case 2: AAT deficiency, hypertension, chronic kidney disease, skin rash, short stature	Case 1: Irritability, lethargy, decreased left arm movement, seizures Case 2: Poor weight gain, vomiting, erythema multiforme, weakness, hypoglycemia, facial droop, stroke	Case 1: Progressed over months Case 2: Developed progressively over months	Case 1: Urticarial skin rash evolving into plaques, hematuria, nephrotic proteinuria, mild LV hypertrophy Case 2: Erythema multiforme, left arm and leg weakness, facial droop, short stature	CBC, liver enzymes, MRI/MRA	Case 1: Elevated aminotransferases, liver biopsy showed fibrosis, renal artery stenosis, nephrotic syndrome, normal creatinine Case 2: Elevated aminotransferases, AATD (alpha-1 antitrypsin deficiency), biallelic SERPINA1 mutations	Case 1: MRI/MRA: Multiple infarctions in different territories (right hemispheric, MCA, PCA). Severe narrowing of arteries (ICA, MCA, ACA). Numerous collateral vessels Case 2: MRI/MRA: Right parieto-occipito-temporal stroke, severe narrowing of bilateral distal ICAs, MCAs, ACAs, chronic infarct with encephalomalacia and gliosis

Abbreviations: ACA, anterior cerebral artery; CT, computed tomography; EDAS, encephaloduroarteriosynangiosis; EEG, electroencephalogram; GH, growth hormone; GHD, growth hormone deficiency; ICA, internal carotid artery; ICH, intracerebral hemorrhage; IGF-1, insulin-like growth factor 1; MCA, middle cerebral artery; MOPD2, microcephalic osteodysplastic primordial dwarfism type II; MRA, magnetic resonance angiography; MRI, magnetic resonance imaging; SAH, subarachnoid hemorrhage; SDS, standard deviation score; SIOD, Schimke immuno-osseous dysplasia; TIA, transient ischemic attack.

**Table 2:** Quality assessment of case reports using CARE guidelines.

**ID**	**Author**	**Patient information**	**Clinical findings**	**Diagnostic assessment**	**Therapeutic interventions**	**Follow-up outcomes**	**Discussion/conclusions**	**Overall quality**
1	Eslava et al. [9]	Comprehensive	Detailed	Thorough	Well documented	Reported	Relevant	High
2	Ishiguro et al. [10]	Comprehensive	Detailed	Thorough	Well documented	Reported	Relevant	High
3	Schuster and Roberts [11]	Comprehensive	Detailed	Thorough	Well documented	Reported	Relevant	High
4	Kalina et al. [12]	Comprehensive	Detailed	Thorough	Well documented	Reported	Relevant	High
5	Lo et al. [13]	Comprehensive	Detailed	Thorough	Not well documented	Reported	Relevant	Moderate
6	Manjila et al. [14]	Comprehensive	Detailed	Thorough	Not documented	Not reported	Needs improvement	Low
7	Choi et al. [15]	Comprehensive	Detailed	Thorough	Well documented	Reported	Relevant	High
8	Yamashita et al. [16]	Comprehensive	Detailed	Thorough	Well documented	Reported	Relevant	High
9	Sravya et al. [17]	Comprehensive	Detailed	Thorough	Well documented	Reported	Relevant	High
10	MacKenzie et al. [18]	Comprehensive	Detailed	Thorough	Well documented	Reported	Needs improvement	Moderate
11	Byard [19]	Comprehensive	Detailed	Thorough	Not documented	Not reported	Needs improvement	Low
12	Nishumora et al. [20]	Comprehensive	Detailed	Thorough	Well documented	Reported	Needs improvement	Moderate
13	Aljthalin et al. [21]	Comprehensive	Detailed	Thorough	Well documented	Reported	Needs improvement	Moderate
14	Young et al. [22]	Comprehensive	Detailed	Thorough	Not documented	Not reported	Needs improvement	Low
15	Hervé et al. [5]	Comprehensive	Detailed	Thorough	Well documented	Reported	Relevant	High
16	Govender et al. [23]	Comprehensive	Detailed	Thorough	Well documented	Reported	Relevant	High
17	Bang et al. [24]	Comprehensive	Detailed	Thorough	Not documented	Not reported	Needs improvement	Low
18	Kato et al. [25]	Comprehensive	Detailed	Thorough	Not documented	Not reported	Needs improvement	Low
19	Vujic et al. [26]	Comprehensive	Detailed	Thorough	Well documented	Reported	Relevant	High
20	Abdullah et al. [27]	Comprehensive	Detailed	Thorough	Well documented	Reported	Needs improvement	Moderate
21	Gburek-Augustat et al. [28]	Comprehensive	Detailed	Thorough	Not documented	Not reported	Needs improvement	Low
22	Kilic et al. [29]	Comprehensive	Detailed	Thorough	Well documented	Reported	Relevant	High
23	Spengos et al. [30]	Comprehensive	Detailed	Thorough	Not documented	Not reported	Needs improvement	Low
24	Strong et al. [31]	Comprehensive	Detailed	Thorough	Well documented	Reported	Relevant	High

**Table 3:** Management plan and outcome.

**ID**	**Author**	**Medical management**	**Surgical management**	**Complications**	**Management of complications**	**Follow-up duration**	**Outcome**	**Mortality rate**
1	Eslava et al. [9]	Follow-up by endocrinology, neurology, and nephrology	NA	NA	NA	NA	Final height < 110 cm	NA
2	Ishiguro et al. [10]	Low-dose aspirin	Under consideration	High fever, erythema, bilateral leg wheels 10 days post aspirin	Stop drugs	4 months	Repeated TIA when aspirin stopped after 4 months	NA
3	Schuster and Roberts [11]	NA	Bilateral EDAS procedure separated by 1-week interval	NA	NA	2 years	No repeated TIA, follow-up for her aortic coarctation, bilateral collaterals in the temporal regions	NA
4	Kalina et al. [12]	Recombinant human GH	Orchidopexy	NA	NA	1 year + continuous follow-up	Improvement in height velocity (12 cm)	NA
5	Lo et al. [13]	Growth hormone	NA	NA	NA	From 3 to 14 years	NA	NA
6	Manjila et al. [14]	No treatment						
7	Choi et al. [15]	Aspirin, calcium channel blockers	Encephaloduroarteriosynangiosis surgery planned	NA	NA	NA	NA	NA
8	Yamashita et al. [16]	Aspirin, pimozide, GH	NA	NA	NA	2 years	No aggravation of moyamoya-like vascular changes	NA
9	Sravya et al. [17]	GH	NA	NA	NA	1 year and 3 months and advised for regular follow-up	No adverse effects	NA
10	MacKenzie et al. [18]	GH and thyroxine Carbamazepine added for the seizure	Exploratory surgery for diagnosis	NA	NA	18 years	9.8-cm velocity growth in a year	NA
11	Byard [19]	NA	NA	NA	NA	NA	NA	Death
12	Nishumora et al. [20]	No treatment mentioned						
13	Aljthalin et al. [21]	Levothyroxine, hydrocortisone, and GH	Right encephaloduroarteriosynangiosis	NA	NA	2 years	Normalized hormonal assay	NA
14	Young et al. [22]	No treatment mentioned						
15	Hervé et al. [5]	NA	NA	NA	NA	NA	NA	Death
16	Govender et al. [23]	Albumin, steroids, ACEi, bisphosphonate, sodium valproate	Poor candidate for surgery	NA	NA	NA	NA	Death
17	Bang et al. [24]	No treatment						
18	Kato et al. [25]	NA	Encephaloduroarteriosynangiosis with the right superficial temporal artery	NA	NA	10 months	No further attacks	NA
19	Vujic et al. [26]	Carbamazepine, aspirin, warfarin, dipyridamole	Not suitable for a revascularization surgery	Subdural hematoma	Aspirin and warfarin stopped	NA	NA	NA
20	Abdullah et al. [27]	GH	NA	NA	NA	6 months	Gained 4 cm height and the other patient 3.5 cm	NA
21	Gburek-Augustat et al. [28]	No treatment						
22	Kilic et al. [29]	Aspirin and physical therapy, enoxaparin, carbamazepine	NA	NA	NA	NA	NA	NA
23	Spengos et al. [30]	No treatment						
24	Strong et al. [31]	1: Aspirin and palliative management 2: Aspirin	2: Bilateral pial synangiosis surgery	NA	NA	NA	NA	1: Death

## Data Availability

All data generated or analyzed during this study are included in published articles.
